# Photochemical Action Plots Reveal the Fundamental Mismatch Between Absorptivity and Photochemical Reactivity

**DOI:** 10.1002/advs.202306014

**Published:** 2023-11-08

**Authors:** Sarah L. Walden, Joshua A. Carroll, Andreas‐Neil Unterreiner, Christopher Barner‐Kowollik

**Affiliations:** ^1^ School of Chemistry and Physics, Centre for Materials Science Queensland University of Technology (QUT) 2 George Street Brisbane QLD 4000 Australia; ^2^ Institute of Solid State Physics and Institute of Applied Physics Abbe Centre of Photonics Friedrich Schiller University Jena Helmholtzweg 3 07743 Jena Germany; ^3^ Institute of Physical Chemistry Karlsruhe Institute of Technology (KIT) Fritz‐Haber‐Weg 2 76131 Karlsruhe Germany; ^4^ Institute of Nanotechnology (INT) Karlsruhe Institute of Technology (KIT) Hermann‐von‐Helmholtz‐Platz 1 76344 Eggenstein‐Leopoldshafen Germany

**Keywords:** absorption, action plot, photochemistry, photophysics, wavelength‐dependent reactivity

## Abstract

Over the last years, the authors' laboratory has employed monochromatic tuneable laser systems to reveal a fundamental mismatch between the absorptivity of a chromophore and its photochemical reactivity for the vast majority of covalent bond forming reactions as well as specific bond cleavage reactions. In the general chemistry community, however, the long‐held assumption pervades that effective photochemical reactions are obtained in situations where there is strong overlap between the absorption spectrum and the excitation wavelength. The current Perspective illustrates that the absorption spectrum of a molecule only provides information about electronic excitations and remains entirely silent on other energy redistribution mechanisms that follow, which critically influence photochemical reactivity. Future avenues of enquiry on how action plots can be understood are proposed and the importance of action plots for tailoring photochemical applications with never‐before‐seen precision is explored.

## Graphical abstract

Absorptivity and photochemical reactivity of molecules do not align in the majority of cases, evidenced by photochemical action plots for bond forming and cleaving reactions. Wavelength resolved action plots capture the sum of all photochemical effects and are the key tool for designing precision orthogonal, synergistic, cooperative and antagonistic photochemical systems in applications ranging from biology to additive manufacturing.

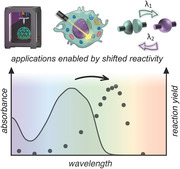

## 1 Introduction to Action Plot Methodology

Photochemistry is the basis of life on earth. Without photosynthesis, life would simply not exist on our planet. Photosynthesis is enabled by broadband light absorption by the critical chromophore chlorophyll. We typically measure the ability of a chromophore to absorb light in an absorption spectrum, revealing information about the electronic excited states. Over the decades, chemists and physicists alike have pondered the question of how to design effective photochemical processes, that is, photochemical reactions with a high yield of the desired target product, and to understand how degradation of materials proceeds under the influence of light, including the degradation of biologically relevant molecules such as DNA.^[^ ^1^, ^2^ ^]^ For chemical structures as vital to life as DNA and chlorophyll, the photoresponse as a function of wavelength was intensively investigated and well‐understood decades ago.^[^ ^3^, ^4^ ^]^ In recent years attention has shifted, and the focus of wavelength dependent studies has been centered around semiconductor photocatalysis^[^ ^5^, ^6^, ^7^ ^]^ and photocurrent generation.^[^ ^8^, ^9^ ^]^ Surprisingly, however, this trend has been largely ignored by materials scientists and the wider chemistry communities. Within these fields, the careful wavelength‐by‐wavelength mapping of photochemical reactivity, both of covalent bond forming and breaking reactions, has not been explored to any significant extent–or even identified as an important avenue of enquiry. Instead, absorption spectra are primarily used as the almost exclusive informant of photochemical reactivity. We note of course that there are fields within (physical) chemistry that provide critical (time‐resolved) insights into energy redistribution after photon absorption for specific molecules. However, they do not examine the wavelength‐by‐wavelength resolved photochemical conversion of a specific reaction on a global level.

Routinely, the employed wavelength is matched with the absorption maximum of the UV‐visible (UV‐Vis) spectrum of a photochemically reactive substrate, following the rationale that the more photons are absorbed, the faster the reaction will progress. In the current Perspective, we dispel the above assumption. For this purpose, we initially introduce our action plot methodology for bond formation of covalent reactions including its surprising findings. We subsequently explore the critical impact of the mismatch between absorptivity and reactivity on practical applications and subsequently move to review the process of light absorption during photochemical reactions, while concomitantly tendering hypotheses for the mismatch between absorptivity and reactivity.

**Figure 1:**
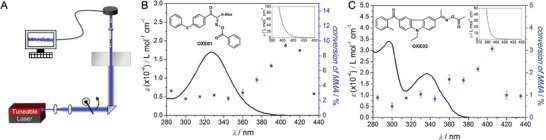
A) Schematic of our action plot apparatus where a tuneable, monochromatic light source is directed from below into a transparent vial containing the reaction solution. The energy incident upon the sample is carefully tuned to ensure an identical photon input at each wavelength of interest. B,C) Comparison of the UV‐vis spectra (insets: zoom into 375/390 to 450 nm) and the wavelength‐dependent conversion of methyl methacrylate initiated by two oxime‐based free‐radical photoinitiators at constant photon count (60 µmol) at each irradiation wavelength. Adapted with permission.^[^ ^10^ ^]^ Copyright 2017, American Chemical Society (ACS).

Several years ago, our team in collaboration with the team of Georg Gescheidt, saw a critical need for wavelength‐by‐wavelength exploration of photochemical reaction systems. This investigation was driven by our serendipitous finding of two photoinitiators for radical polymerization that appear to only absorb < 400 nm, but can induce highly effective macromolecular chain growth when irradiated with visible light.^[^ ^10^ ^]^ Indeed, not only can these two initiators initiate in the visible light range, but their peak performance is significantly red‐shifted compared to their absorbance spectrum measured in the reaction solution (**Figure** **1**). This is a critical finding that at the time received only limited attention in the following literature, except by our team, and was met with incredulity at conferences. The data shown in Figure 1 was obtained via our so‐called “action plot methodology”, using a nanosecond pulsed, wavelength‐tunable laser system capable of delivering an identical and stable number of photons at each wavelength. Typically, a stock solution of the photoreactive compound (or reaction mixture) is divided into aliquots that are independently subjected to monochromatic light. The yield (or conversion) of the photochemical process under examination is subsequently determined by a suitable sensor such as (gravimetrically determined) conversion, change in UV‐Vis absorption or nuclear magnetic resonance frequency changes. For a detailed description of our action plot methodology, the reader is referred to the “How To” instructions provided in the supporting information section of a recent technical overview.^[^ ^11^ ^]^

Over the past years, we have recorded a large number of action plots on a variety of photochemical reaction systems, and we refer the reader to the above noted summary. Pleasingly, other laboratories have since corroborated our findings, predominantly in the space of (controlled) photopolymerization and mostly employing LEDs with narrow emission profiles.^[^ ^12^, ^13^, ^14^, ^15^ ^]^ For the purposes of our current perspective, we will focus on specific examples to illustrate key points, explore important implications that these findings have for applications, and reflect upon possible reasons why we observe such paradigm‐changing disparities between absorptivity and reactivity.

## 2 Implications for Applications

Identifying the most efficient irradiation wavelengths for photoactive groups is of paramount importance in order to judiciously design materials for a desired application. This includes a plethora of everyday examples, ranging from the light driven curing of dental fillings to the fabrication of coatings. If the optimum wavelength is known, the photochemical reactions underpinning curing applications can progress faster and to higher conversions, reducing waste and increasing economic advantages. Imagine a situation where the initiators depicted in Figure 1 were excited in a curing formulation at 330 nm, selected based on the absorption spectrum, instead of at the most effective wavelength of 420 nm indicated by the action plot.

**Figure 2:**
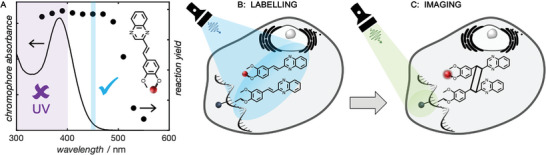
A) Action plot of styrylquinoxaline in water indicating strongly red‐shifted reactivity into the blue and green light regions and B) schematic diagram of the application of 455 nm blue light induced [2 + 2] cycloaddition of styrylquinoxaline for DNA labeling and C) fluorescence readout. a) Adapted with permission.^[^ ^16^ ^]^ Copyright 2020, Springer Nature. b,c) Adapted with permission.^[^ ^17^ ^]^ Copyright 2023, Royal Society of Chemistry.

In the realm of biological applications, efficient chemistries in the biologically benign window are essential. Here, action plots can provide key guidance too. As highlighted in the current contribution, our action plot studies have uncovered unexpected reaction windows that are often red‐shifted relative to the absorption maximum. Such measurements on existing chromophores, which may have previously been ruled out for biological applications due the short wavelength absorption maximum, could in fact be found to be safe and efficient in longer wavelength regimes. One powerful example is the styrylquinoxaline chromophore depicted in **Figure** **2**, which can undergo an efficient [2 + 2] cycloaddition at excitation wavelengths up to 500 nm, despite having an absorption maximum at 380 nm and seemingly no absorption above ≈480 nm.^[^ ^16^ ^]^ Inspired by these findings, Michenfelder et al. recently exploited styrylquinoxaline for DNA labeling with a mild 450 nm LED.^[^ ^17^ ^]^ The power of the action plot in this case was to reveal the extent of the molecule's reactivity in the long wavelength region. Without these measurements, and using the absorption spectrum as the sole guide, one would assume there would be minimal dimerization of the styrylquinoxaline at wavelengths longer than 450 nm – ignoring potential DNA labeling in the green region of the visible light spectrum. Similar biological labeling has been reported with 455 nm light using styrylpyrene as the labeling moiety; considerably red‐shifted from its absorption maximum ≈380 nm.^[^ ^18^, ^19^ ^]^ We predict that as the wavelength dependence of more biologically compatible chromophores are investigated, more visible and NIR active molecules will be unearthed.

The commonly observed bathochromic shift in reactivity also has benefits for broader materials applications. As many organic polymers absorb light in the UV region, the development of visible‐light tuneable chromophores has some obvious advantages. These include higher penetration depths into the material, benign irradiation conditions and the potential for orthogonal activation. One such example is the well‐known case of anthracene dimerization, which is commonly believed to only occur under UV excitation.^[^ ^20^, ^21^ ^]^ A detailed action plot study, however, revealed that the dimerization can indeed proceed under much milder visible light irradiation up to 410 nm.^[^ ^22^ ^]^ This long wavelength dimerization facilitated the incorporation of anthracene into 3D printed structures, and the subsequent mechanical tuning of structures with blue light.^[^ ^23^ ^]^ Wavelength dependent, dynamic materials have also been developed based on the exchange of disulphide and diselenide bonds.^[^ ^24^ ^]^ At short wavelengths, the disulphide and diselenide bonds undergo metathesis, effectively exchanging bonds with each other to generate a crosslinked network, while long wavelength irradiation induces a reversion back to the original bond structure. Combining these wavelengths' orthogonal behavior permits dynamic and robust bond formation and cleavage. The development of more materials with mild synthetic stimuli, as well as degradation stimuli, are critical to tackle challenges such plastic recycling. To this end, Do et al. demonstrated a polymer ligation system where UVA light was used to polymerise a multicomponent polymer and shorter wavelength UVB light was later applied for degradation.^[^ ^25^ ^]^

**Figure 3:**
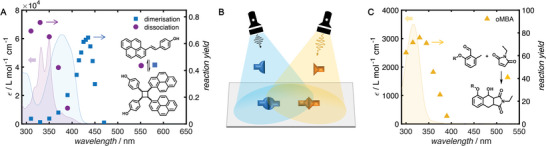
Example of two‐color photochemistry. A) Action plot measurements on the cycloaddition and cycloreversion of styrylpyrene highlighting that dissociation supresses the dimerization conversion at short wavelengths.^[^ ^28^ ^]^ B) Schematic diagram of orthogonal activation of two chromophores in a single photoresist, in this case the two chromophores are styrylpyrene and *o*‐methylbenzaldehyde (oMBA). C) Action plot measurements of oMBA reaction with *N*‐ethylmaleimide showing efficient reaction in the wavelength region where styrylpyrene dimerization is supressed.^[^ ^29^ ^]^

**Figure 4:**
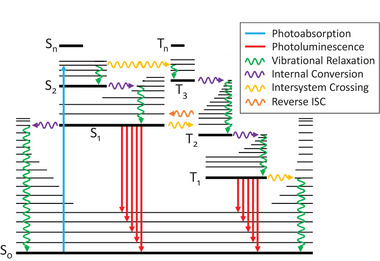
Jablonski diagram illustrating molecular excitation through absorption of a single photon to a specific vibrational energy. Radiative and non‐radiative pathways exist for the return to the ground state. The energy levels, along with available pathways and their propensities, are unique to each molecule. Singlet states from ground (S_0_) to states higher (S_n_) increase in energy, while T_n_ represents triplet states. Each band corresponds to a vibrational energy level within that molecular state. Rotational energies are not shown for each vibrational energy.

Action plots are perhaps even more critical where two colors of light are required to facilitate a photochemical reaction, such as in emerging 3D printing technologies. Here, they are essential to determine the specific wavelengths where individual chromophores, or synthetic steps, can be addressed individually for synergistic, orthogonal, antagonistic or sequential purposes.^[^ ^26^ ^]^ Previously, our group exploited an orthogonal chemical system to show that disparate material properties can be produced from a single photoresist purely by applying different colors of light;^[^ ^27^ ^]^ a feat that could only be achieved with the insights available from action plot measurements. The action plots depicted in **Figure** **3** were particularly important for this result, as the absorption spectra indicated that both molecules, styrylpyrene and *o*‐methylbenzaldehyde, absorb light at shorter (UV) wavelengths.^[^ ^28^, ^29^ ^]^ It was only through the discovery of a narrow wavelength region in the UV where cycloreversion of the styrylpyrene dimer dominated over cycloaddition (**Figure** **4**a) that orthogonal addressability was realized.

Since the above initial example, we have further developed two‐color, synergistically activated photoresists, where the individual colors activate individual components of the photoresist. Only at the intersection of both colors of light, can photocuring occur.^[^ ^27^, ^30^ ^]^ Similar two‐color photocuring resists have also been developed by the groups of Hecht and Wegener, in the latter case using 2‐step photoinitiator activation, where each step is activated by a different color of light.^[^ ^31^, ^32^ ^]^ Examples of such two‐color photoresists are rare, and hence the applications for so‐called xolography or light‐sheet printing remain a field with rich development opportunities. There appears to be scope for many other, as yet undiscovered, compatible molecular pairs capable of orthogonal activation to emerge. As more photoinitiators and resists are developed, broader applications, such as conductive or biologically compatible 3D printed materials will similarly expand.

## 3 The Mismatch between Absorptivity and Photochemical Reactivity

While a complete understanding of the mechanism for the mismatch between absorptivity and reactivity remains elusive, some attempts have been made at rationalizing it. In the following, we will discuss the process of absorption of light by a molecule, while concomitantly highlighting key experiments in the literature that investigate explanations for the mismatch. It is important to note that most photochemical systems are deeply complex and that a range of different mechanisms may all contribute to varying degrees.

Several foundational principles have been developed to aid in understanding photochemical reactions, inform predictions of the potential products formed and gauge the efficacy with which they are generated. Fermi's Golden Rule is one such principle, which states that “the stronger the overlap in the wavefunctions of the respective electronic states, the higher the probability of transition and hence the transition rate”.^[^ ^33^ ^]^ Experimentally, this manifests as the order of magnitude of the extinction coefficient in Beer‐Lambert's law, quantifying the transition from the electronic ground state to an excited state. Furthermore, it is responsible for the profile of an absorbance spectrum. It is important to note that absorption is merely the first step of many on the path of a photochemical reaction, and it is therefore no surprise that consultation of an absorbance spectrum alone may not provide the entire information for every molecular system. Experienced photochemists may understand this fact well, however without a strong understanding of photochemistry it can be easy to rely solely on an absorbance spectrum to choose an efficient excitation wavelength. Other principles focus on light emission, including Kasha's Rule which states that “polyatomic molecules react or luminesce only from the lowest excited state of each multiplicity”,^[^ ^34^ ^]^ and Vavilov's Rule that states that “the luminescence quantum yield is independent of excitation wavelength”.^[^ ^35^ ^]^ The naïve interpretation of these rules is that molecules should react at appreciable yields only from the lowest lying levels of the singlet and triplet states and that these yields should also be excitation‐independent. This interpretation implies that the absorption spectrum should seemingly dictate the reactivity spectrum for molecules that strictly obey these two rules. Again, experienced photochemists know there are many exceptions to these rules, yet their very existence as rules suggests that they govern most molecular systems, and if taken as absolute can further reinforce incorrect assumptions that an absorption spectrum is sufficient to determine reactivity for all molecular systems.

At this point it is useful to transition from discussions of principles and theory and determine exactly what empirical information an absorption spectrum provides about a system, to better comprehend its limitations. The most valuable information obtained from absorbance spectra is the energy levels for singlet excitations and, in much rarer cases, triplet excitations that are directly accessible from the ground state.^[^ ^36^, ^37^ ^]^ The magnitude of absorption peaks reveals the relative likelihood of a transition occurring at each excitation wavelength, while the width of each peak indicates the range of vibrational and rotational energies for that specific excited state – as well as contributions from solvent interactions.^[^ ^38^ ^]^ Qualitative insight into the rates of internal conversion (IC) can also be obtained from the widths of absorption peaks. However, gaining quantitative information such as lifetimes and specific relaxation pathways, requires experimental methods such as ultrafast time‐resolved spectroscopy.^[^ ^39^ ^]^ Based on these insights into absorption, several explanations between reactivity and an absorbance spectrum have been put forth, including light attenuation by Beer‐Lambert's law at high extinction coefficients, and the possibility of multi‐photon absorption. While variations in sample concentration contribute,^[^ ^29^ ^]^ Beer‐Lambert's law cannot be the sole contributor, as if it were, the action plot would be symmetric around the absorption peak, which is rarely the case.^[^ ^40^ ^]^ In addition, even wavelength dependent surface ligations on a single atomic monolayer still reveal a weak bathochromic shift.^[^ ^41^ ^]^ With regard to two photon processes, action plot experiments performed with LEDs as the excitation source also display a similar mismatch between absorption and reaction yields.^[^ ^14^, ^15^, ^42^ ^]^ These results effectively rule out the possibility of a multi‐photon mechanism as the cause of the bathochromic shifts. Changes in molecular absorbance during irradiation were also disregarded as a mechanism, with continuous monitoring of an absorbance spectrum during irradiation showing no increase in absorbance in the longer wavelength region, while still showing enhanced reactivity at these excitations.^[^ ^43^ ^]^ Could violations of Kasha's Rule be responsible for all the observed shifts? With so many molecular systems studied, and most of them showing discrepancies between the absorption and reactivity maxima, it seems more likely that there is still some yet unrealized mechanism at work.

To further explore the mismatch between absorption and reactivity, we must go beyond the absorption spectrum and interrogate the steps following absorption.^[^ ^44^ ^]^ A molecule that is excited to a certain electronic state has access to several available pathways, illustrated in a Jablonski diagram (Figure 4). Energy relaxation commences with non‐radiative decay, such as vibrational relaxation and IC, which describe transitions between vibrational states of the same or overlapping electronic states, respectively. Vibrational relaxation facilitates energy dissipation through vibrational, translational and conformational relaxation during collisions with surrounding molecules, or as intramolecular vibrational redistribution (IVR) where the energy is redistributed amongst other vibrational modes within the same molecule. When the vibrational levels of the singlet excited state overlap with a triplet state, intersystem crossing (ISC) can occur, which is the radiationless transition between singlet and triplet electronic states, featuring different spin multiplicities. Triplet lifetimes typically exceed those of singlet states, making triplet states particularly important for many chemical reactions. ISC can also occur in reverse, known as reverse‐ISC. All the processes mentioned thus far are radiationless, with the excess energy dissipating in the form of heat. Radiative pathways also exist which emit the excess energy via a luminescent process, either fluorescence from a singlet state, or phosphorescence from a triplet state. Finally, the energy can also be directly transferred between molecules, such as from a donor to an acceptor through mechanisms such as Förster resonance energy transfer,^[^ ^45^, ^46^ ^]^ Dexter Energy Transfer,^[^ ^47^ ^]^ (including Triplet‐Triplet Annihilation),^[^ ^48^ ^]^ or as a proton transfer process.^[^ ^49^ ^]^

Now we have outlined all the relevant processes, we consider two broad conditions that are critical for a successful photochemical reaction. First, access to a favorable reaction pathway, and second, a sufficiently long excited state lifetime for the reaction to occur. For the first condition, exceptions to Kasha's rule are critical, for in these cases the absorption spectrum reveals excited states that may not be photochemically active.^[^ ^50^ ^]^ Furthermore, efficient ISC to a reactive triplet state relies on sufficient overlap between singlet and triplet vibrational energy levels. This vital information is not revealed in the absorption spectra and can directly contribute to wavelength dependent variations in reactivity. Experiments on photoluminescence of a low‐temperature indole solution have shown that the ratio of fluorescence to phosphorescence changes with longer wavelengths.^[^ ^51^ ^]^ This effect arose due to interactions between the solvent and dipole moments of two closely aligned singlet electronic excitations. As both states had differing magnitudes in dipole moment, their interactions with the molecules of a polar solvent were found to be separate, resulting in a dependence on ISC quantum yields from excitations at the red edge of the absorption spectrum.^[^ ^52^ ^]^ If these interactions lead to more efficient ISC at lower energy excitations for a given chromophore, we suggest this process could contribute to observed red‐shifted action spectra. Reeves et al. recently reported a Norrish Type 1 photoreaction displaying the fastest conversion utilizing a wavelength that is red‐shifted from the absorbance spectrum.^[^ ^15^ ^]^ These authors proposed that the reaction is less efficient from higher electronic states excited with UV light, as the ISC pathway to T_1_ from S_1_ is potentially more efficient than from S_2_ or S_n_, but noted that femtosecond transient absorption spectroscopy was required to underpin this hypothesis.^[^ ^54^ ^]^

The second condition, regarding excited state lifetimes, becomes particularly significant in bimolecular reactions. Consider processes such as cycloaddition or donor‐acceptor reactions, where two or more molecules must be in close proximity. Here, longer excitation lifetimes are beneficial, implying a higher chance of these excited molecules experiencing collisions prior to electron relaxation. For these molecular classes, an excitation wavelength that ultimately leads to a long‐lived triplet state will likely result in a more efficient reaction than wavelengths leading to short‐lived singlet excitations.^[^ ^53^, ^54^ ^]^

We finish by discussing the contributions of specific transitions to the reactivity mismatch, starting with the pathways involving IC and IVR. In the absence of competing photo‐induced processes (i.e., following the IC path from S_1_ to S_o_ in Figure 4, purple arrow to the left) and depending on the excited state lifetime, the molecule arrives vibrationally hot in the electronic ground state. Interestingly, high‐temperature spectra of molecules obtained in the gas phase under high‐temperature shock tube conditions clearly display red‐shifts^[^ ^55^ ^]^ and their spectral width is also wider. Thus, transiently higher extinction coefficients are possible in the blue region as well.^[^ ^56^ ^]^ Why does this matter for experiments performed with LEDs or nanosecond lasers? Typically, molecules require a few to hundreds of picoseconds to dissipate energy after photo‐excitation. Similar to shock tubes, LEDs (or nanosecond lasers) constantly re‐pump the relaxing system back into higher states eventually maintaining a much higher temperature compared to ambient conditions. Thus, a “hot‐spectra” hypothesis may be tendered as a potential contributing factor for the explanation of our observed red‐shifted action spectra. Finally, there are other transitions that may contribute and must be considered, that is, strong vibrational coupling may play an essential role in the energy dissipation process prior to chemical reactions as it can lead to a tilting of the reactivity landscape.^[^ ^57^, ^58^ ^]^ All these processes are invisible to steady‐state methods such as an absorbance spectrum and therefore require more sophisticated techniques to disentangle them.

With so many available chromophores, in so many unique systems, it is thus critically important to avoid solely relying upon absorbance spectra for determining reaction efficiencies. Through a wavelength‐tunable laser system and our action plot methodology, these complex underlying mechanisms can be holistically observed as a global effect. From a practical point of view, action plots are thus the only reliable methodology to access comprehensive wavelength dependent reactivity information. The underlying reasons for the observed disparities require careful, separate consideration and are certainly system‐specific and multi‐causal. However, it is equally true that nearly all investigated photochemical systems show a marked departure from their absorption spectra in terms of their reactivity. Thus, it is not unreasonable to assume that there is one overarching guiding principle at work that causes the typical strong red‐shifts.

## 4 Summary and Outlook

In the current perspective, we have highlighted the now accepted paradigm that absorption spectra are not a reliable guide for selecting optimum wavelengths to induce photochemical reactions. We have considered the reasons why this should not come as a surprise, although the reliance on absorption spectra for predicting reactivity continues to be ongoing practice in chemistry, despite overwhelming evidence to the contrary. Recording photochemical action plots for covalent bond forming and breaking photochemical reactions is an effective means for capturing the sum of all effects governing a photochemical process, with key applications in a wide range of fields. Action plot measurements facilitate photochemistry with never‐before‐seen precision, as reactivity windows can be determined for each individual reaction system, enabling advanced concepts including wavelength orthogonal, synergistic, cooperative and antagonistic photochemical reactions. Unpacking the sum of all processes that an action plot affords for a particular photochemical process is a complex undertaking, with each system requiring careful individual consideration. Techniques that can assist include transient absorption spectroscopy as well as quantum chemical calculations. We submit, however, that action spectra will – from a practical perspective – remain the only broadly applicable avenue to gain insights into photochemical reactivity. There is a vast array of photochemical reaction systems that are yet to be subjected to action plot analysis. It will be fascinating to see a fine wavelength resolved image of photochemical reactivity emerge over the next years, as more research groups adapt the technology to design precision photochemical system that find applications from biology to material science.

## Conflict of Interest

The authors declare no conflict of interest.

## References

[1] G. P. Pfeifer , Y.‐H. You , A. Besaratinia , Mutat Res 2005, 571, 19. 15748635 10.1016/j.mrfmmm.2004.06.057

[2] S. Madronich , R. L. Mckenzie , L. O. Björn , M. M. Caldwell , J. Photochem. Photobiol., B 1998, 46, 5. 9894350 10.1016/s1011-1344(98)00182-1

[3] C. G. B. X. Daubeny , Philos. Trans. R. Soc. London 1997, 126, 149.

[4] R. B. Setlow , Proc. Natl. Acad. Sci. USA 1974, 71, 3363. 4530308 10.1073/pnas.71.9.3363 PMC433772

[5] S.‐K. Lee , A. Mills , C. O'rourke , Chem. Soc. Rev. 2017, 46, 4877. 28665437 10.1039/c7cs00136c

[6] E. Kowalska , R. Abe , B. Ohtani , Chem. Commun. 2009, 0, 241. 10.1039/b815679d 19099082

[7] X. Yan , T. Ohno , K. Nishijima , R. Abe , B. Ohtani , Chem. Phys. Lett. 2006, 429, 606.

[8] L. A. A. Pettersson , L. S. Roman , O. Inganäs , J. Appl. Phys. 1999, 86, 487.

[9] R. Murdey , N. Sato , in Advances in Organic Crystal Chemistry, (Eds: R. Tamura , M. Miyata ), Springer, Tokyo 2015.

[10] D. E. Fast , A. Lauer , J. P. Menzel , A.‐M. Kelterer , G. Gescheidt , C. Barner‐Kowollik , Macromolecules 2017, 50, 1815.

[11] I. M. Irshadeen , S. L. Walden , M. Wegener , V. X. Truong , H. Frisch , J. P. Blinco , C. Barner‐Kowollik , J. Am. Chem. Soc. 2021, 143, 21113. 34859671 10.1021/jacs.1c09419

[12] J. Zhang , J. Lalevée , N. S. Hill , K. Launay , F. Morlet‐Savary , B. Graff , M. H. Stenzel , M. L. Coote , P. Xiao , Macromolecules 2018, 51, 8165.

[13] R. W. Hughes , M. E. Lott , J. I. Bowman , Brent S. Sumerlin , ACS Macro Lett. 2023, 12, 14. 36533885 10.1021/acsmacrolett.2c00683

[14] C. Ma , T. Han , S. Efstathiou , A. Marathianos , H. A. Houck , D. M. Haddleton , Macromolecules 2022, 55, 9908. 36438594 10.1021/acs.macromol.2c01413 PMC9686136

[15] J. A. Reeves , N. De Alwis Watuthanthrige , C. Boyer , D. Konkolewicz , ChemPhotoChem 2019, 3, 1171.

[16] K. Kalayci , H. Frisch , V. X. Truong , C. Barner‐Kowollik , Nat. Commun. 2020, 11, 4193. 32826921 10.1038/s41467-020-18057-9 PMC7443129

[17] R. T. Michenfelder , L. Delafresnaye , V. X. Truong , C. Barner‐Kowollik , H.‐A. Wagenknecht , Chem. Commun. 2023, 59, 4012. 10.1039/d3cc00817g 36920883

[18] S. Bialas , L. Michalek , D. E. Marschner , T. Krappitz , M. Wegener , J. Blinco , E. Blasco , H. Frisch , C. Barner‐Kowollik , Adv. Mater. 2019, 31, 1807288. 10.1002/adma.201807288 30614578

[19] T. Doi , H. Kawai , K. Murayama , H. Kashida , H. Asanuma , Chemistry 2016, 22, 10533. 27299696 10.1002/chem.201602006

[20] H. Bouas‐Laurent , J.‐P. Desvergne , A. Castellan , R. Lapouyade , Chem. Soc. Rev. 2000, 29, 43.

[21] J. Van Damme , F. Du Prez , Prog. Polym. Sci. 2018, 82, 92.

[22] A. Kislyak , H. Frisch , M. Gernhardt , P. H. M. Van Steenberge , D. R. D'hooge , C. Barner‐Kowollik , Chemistry 2020, 26, 478. 31489724 10.1002/chem.201903641

[23] M. Gernhardt , E. Blasco , M. Hippler , J. Blinco , M. Bastmeyer , M. Wegener , H. Frisch , C. Barner‐Kowollik , Adv. Mater. 2019, 31, 1901269. 10.1002/adma.201901269 31155785

[24] F. Fan , S. Ji , C. Sun , C. Liu , Y. Yu , Y. Fu , H. Xu , Angew. Chem., Int. Ed. 2018, 57, 16426. 10.1002/anie.201810297 30345597

[25] P. T. Do , B. L. J. Poad , H. Frisch , Angew. Chem. 2023, 135, e202213511. 10.1002/anie.202213511 PMC10108003 36535898

[26] J. Hobich , E. Blasco , M. Wegener , H. Mutlu , C. Barner‐Kowollik , Macromol. Chem. Phys. 2023, 224, 2200318.

[27] S. L. Walden , L. L. Rodrigues , J. Alves , J. P. Blinco , V. X. Truong , C. Barner‐Kowollik , Nat. Commun. 2022, 13, 2943. 35618722 10.1038/s41467-022-30002-6 PMC9135712

[28] D. E. Marschner , H. Frisch , J. T. Offenloch , B. T. Tuten , C. R. Becer , A. Walther , A. S. Goldmann , P. Tzvetkova , C. Barner‐Kowollik , Macromolecules 2018, 51, 3802.

[29] J. P. Menzel , B. B. Noble , A. Lauer , M. L. Coote , J. P. Blinco , C. Barner‐Kowollik , J. Am. Chem. Soc. 2017, 139, 15812. 29024596 10.1021/jacs.7b08047

[30] T. N. Eren , F. Feist , K. Ehrmann , C. Barner‐Kowollik , Angew. Chem., Int. Ed. 2023, 62, e202307535. 10.1002/anie.202307535 37358799

[31] M. Regehly , Y. Garmshausen , M. Reuter , N. F. König , E. Israel , D. P. Kelly , C.‐Y.u Chou , K. Koch , B. Asfari , S. Hecht , Nature 2020, 588, 620. 33361791 10.1038/s41586-020-3029-7

[32] V. Hahn , P. Rietz , F. Hermann , P. Müller , C. Barner‐Kowollik , T. Schlöder , W. Wenzel , E. Blasco , M. Wegener , Nature Photonics 2022, 16, 784.

[33] E. C. V. Fermi , Nuclear Physics, University of Chicago Press, Chicago 1950.

[34] M. Kasha , Discuss. Faraday Soc. 1950, 9, 14.

[35] P. Klán , J. Wirz , Photochemistry of Organic Compounds, John Wiley & Sons, Hoboken, NJ 2009.

[36] J. Yuan , R. Chen , X. Tang , Y. Tao , S. Xu , L. Jin , C. Chen , X. Zhou , C. Zheng , W. Huang , Chem. Sci. 2019, 10, 5031. 31183053 10.1039/c8sc05198d PMC6530535

[37] B. F. Minaev , S. Knuts , H. Ågren , Chem. Phys. 1994, 181, 15.

[38] D. C. Harris , C. A. Lucy , Quantitative chemical analysis, 10th ed. Macmillan Learning, Austin 2020.

[39] A. H. Zewail , J. Phys. Chem. A 2000, 104, 5660.

[40] P. W. Kamm , J. P. Blinco , A.‐N. Unterreiner , C. Barner‐Kowollik , Chem. Commun. 2021, 57, 3991. 10.1039/d1cc00340b 33885643

[41] L. Michalek , T. Krappitz , K. Mundsinger , S. L. Walden , L. Barner , C. Barner‐Kowollik , J. Am. Chem. Soc. 2020, 142, 21651. 33337866 10.1021/jacs.0c11485

[42] S. L. Walden , H. Frisch , B. V. Unterreiner , A.‐N. Unterreiner , C. Barner‐Kowollik , J. Chem. Educ. 2020, 97, 543.

[43] I. M. Irshadeen , K. De Bruycker , A. S. Micallef , S. L. Walden , H. Frisch , C. Barner‐Kowollik , Polym. Chem. 2021, 12, 4903.

[44] A. D. Smith , E. M. Warne , D. Bellshaw , D. A. Horke , M. Tudorovskya , E. Springate , A. J. H. Jones , C. Cacho , R. T. Chapman , A. Kirrander , R. S. Minns , Phys. Rev. Lett. 2018, 120, 183003. 29775354 10.1103/PhysRevLett.120.183003

[45] A. Kaur , P. Kaur , S. Ahuja , Anal. Methods 2020, 12, 5532. 33210685 10.1039/d0ay01961e

[46] O. Ostroverkhova , Chem. Rev. 2016, 116, 13279. 27723323 10.1021/acs.chemrev.6b00127

[47] A. Monguzzi , R. Tubino , F. Meinardi , Phys. Rev. B 2008, 77, 155122.

[48] T. N. Singh‐Rachford , F. N. Castellano , Coord. Chem. Rev. 2010, 254, 2560.

[49] L. G. Arnaut , S. J. Formosinho , J Photochem Photobiol A Chem 1993, 75, 1.

[50] K. Zlatic , M. Bogomolec , M. Cindric , L. Uzelac , N. Basaric , Tetrahedron 2022, 124, 132995.

[51] A. A. Sukhodola , Chem. Phys. Lett. 2020, 754, 137674.

[52] A. P. Demchenko , Luminescence 2002, 17, 19. 11816059 10.1002/bio.671

[53] L. D. Elliott , S. Kayal , M. W. George , K. Booker‐Milburn , J. Am. Chem. Soc. 2020, 142, 14947. 32786778 10.1021/jacs.0c05069

[54] T. J. A. Wolf , D. Voll , C. Barner‐Kowollik , A.‐N. Unterreiner , Macromolecules 2012, 45, 2257.

[55] D. C. Astholz , L. Brouwer , J. Troe , Berichte der Bunsengesellschaft für physikalische Chemie 1981, 85, 559.

[56] O. Schalk , J.‐P. Yang , A. Hertwig , H. Hippler , A. N. Unterreiner , Mol. Phys. 2009, 107, 2159.

[57] A. Thomas , L. Lethuillier‐Karl , K. Nagarajan , R. M. A. Vergauwe , J. George , T. Chervy , A. Shalabney , E. Devaux , C. Genet , J. Moran , T. W. Ebbesen , Science 2019, 363, 615. 30733414 10.1126/science.aau7742

[58] T. Brixner , G. Gerber , ChemPhysChem 2003, 4, 418. 12785256 10.1002/cphc.200200581

